# Development of Salivary Cortisol Circadian Rhythm and Reference Intervals in Full-Term Infants

**DOI:** 10.1371/journal.pone.0129502

**Published:** 2015-06-18

**Authors:** Katrin Ivars, Nina Nelson, Annette Theodorsson, Elvar Theodorsson, Jakob O. Ström, Evalotte Mörelius

**Affiliations:** 1 Department of Clinical and Experimental Medicine, Division of Pediatrics, Faculty of Health Sciences, Linköping University, Linköping, Sweden; 2 Department of Pediatrics, Institute of Clinical Sciences, Gothenburg University, Gothenburg, Sweden; 3 Division of Neuroscience, Department of Clinical and Experimental Medicine, Faculty of Health Sciences, Linköping University, Department of Neurosurgery, Anaesthetics, Operations and Specialty Surgery Center, Region Östergötland, Linköping, Sweden; 4 Division of Microbiology and Molecular Medicine, Department of Clinical and Experimental Medicine, Faculty of Health Sciences, Linköping University, Department of Clinical Chemistry, Center for Diagnostics, Region Östergötland, Linköping, Sweden; 5 Department of Social and Welfare Studies, Division of Health, Activity and Care, Faculty of Health Sciences, Linköping University, Linköping, Sweden; Istituto Superiore di Sanità, ITALY

## Abstract

**Background:**

Cortisol concentrations in plasma display a circadian rhythm in adults and children older than one year. Earlier studies report divergent results regarding when cortisol circadian rhythm is established. The present study aims to investigate at what age infants develop a circadian rhythm, as well as the possible influences of behavioral regularity and daily life trauma on when the rhythm is established. Furthermore, we determine age-related reference intervals for cortisol concentrations in saliva during the first year of life.

**Methods:**

130 healthy full-term infants were included in a prospective, longitudinal study with saliva sampling on two consecutive days, in the morning (07:30-09:30), noon (10:00-12:00) and evening (19:30-21:30), each month from birth until the infant was twelve months old. Information about development of behavioral regularity and potential exposure to trauma was obtained from the parents through the Baby Behavior Questionnaire and the Life Incidence of Traumatic Events checklist.

**Results:**

A significant group-level circadian rhythm of salivary cortisol secretion was established at one month, and remained throughout the first year of life, although there was considerable individual variability. No correlation was found between development of cortisol circadian rhythm and the results from either the Baby Behavior Questionnaire or the Life Incidence of Traumatic Events checklist. The study presents salivary cortisol reference intervals for infants during the first twelve months of life.

**Conclusions:**

Cortisol circadian rhythm in infants is already established by one month of age, earlier than previous studies have shown. The current study also provides first year age-related reference intervals for salivary cortisol levels in healthy, full-term infants.

## Introduction

Cortisol is considered a major biomarker of stress among adults, children and infants [1]. The fetal hypothalamic-pituitary-adrenal system responsible for cortisol release is functional from the beginning of the second trimester [2]. Cortisol is secreted in a pulsatile fashion and is known to display a circadian rhythm in adults and older children. Cortisol levels are highest in the morning and subsequently decrease to a nadir in the evening [3]. Infants are thought to develop cortisol circadian rhythm (CCR) during the first year of life, but exactly when has not yet been established. Such knowledge is essential for the potential use of salivary cortisol for diagnostic and research purposes. Several prior studies of full-term infants have investigated development of CCR in salivary cortisol, but have reached different conclusions regarding when CCR is established, varying from two weeks to more than nine months of age [4–10]. There may be a variety of possible explanations for these divergent results: lack of consensus regarding how to define CCR, different sampling times and frequencies, low sample numbers, and a low number of participants (often less than 20, never more than 75).

Previous studies have investigated factors that may influence the development of CCR in infants. Price et al. (1983) found a weak correlation between the evolution of CCR and regularity in sleep (age when infants start to sleep more than six consecutive hours). Different life events, including trauma and depression, have also been shown to influence the hypothalamic-pituitary-adrenal axis, and thereby cortisol secretion and circadian rhythm. An earlier study on adolescents demonstrated that trauma affects the hypothalamic-pituitary-adrenal axis by raising morning cortisol levels, while evening levels remained unchanged [11]. In contrast, multiple traumas lower basal cortisol and flatten the CCR in adults [12].

Salivary cortisol is a feasible parameter for studying the stress response to different interventions early in life [13, 14]. Measuring cortisol in saliva has several advantages over plasma measurements, particularly since collection of saliva does not cause stress or pain for the infant [13, 15]. Furthermore, it has been argued that salivary cortisol is more representative of the biologically active fraction of cortisol, compared with plasma cortisol levels [3]. To the best of our knowledge, reference intervals for salivary cortisol levels in infants during the first year of life have not previously been published. Salivary cortisol reference intervals for healthy, full-term infants up to one year of age are likely to be of value both for future research and in clinical practice to evaluate cortisol levels as expressions of stress or disease.

The aims of the present study are to establish at what age CCR is developed and to investigate if behavioral regularity is related to CCR development, and to establish reference intervals for salivary cortisol in infants.

## Materials and Methods

### Participants

A total of 130 healthy full-term infants born at the University Hospital of Linköping or at Ryhov Hospital in Jönköping were included in the study. Characteristics of the subjects are compiled in Table 1.

**Table 1:** Characteristics of subjects and parents.

Participants	Healthy full-term infants
Total number (n)	130
Girls/Boys (n)	69/61
Gestational age (week)	37+0–42+2
Mothers age (years), mean(SD)	31(4)
Fathers age (years), mean(SD)	33(5)
Firstborn (n)	53
Second born (n)	43
Third born (n)	26
Fourth born (n)	6
Fifth born (n)	1
Mother’s education	
Secondary professional education (n)	22
Secondary academic education (n)	6
College/university education (n)	102
Mother’s occupation	
Employed (n)	98
Parental leave (n)	16
Unemployed (n)	2
Missing answers (n)	14
Father’s education	
Primary education (n)	1
Secondary professional education (n)	28
Secondary academic education (n)	5
College/university education (n)	96
Father’s occupation	
Employed (n)	114
Parental leave (n)	0
Unemployed (n)	1
Missing answers (n)	16
Nationality of parents	
2 Swedish parents (n)	122
1 Swedish parent and1 non-Swedish European parent (n)	4
1 Swedish parent and1 non-Swedish non-European parent (n)	3
2 non-Swedish non-European parents (n)	1

Number of subjects (n), Standard Deviation (SD),

All babies were vaginally delivered after normal pregnancies. At inclusion, they were healthy and of normal weight, height and head circumference. All infants were breastfed at the beginning of the study; for details see Table 2.

**Table 2:** Number of infants; breast milk-fed and formula-fed, during the first year.

Age (month)	Breast milk-fed (n)	Infant formula-fed (n)	No answer (n)
**0**	130	0	0
**1**	123	2	5
**2**	116	11	7
**3**	109	9	12
**4**	104	12	14
**5**	93	19	18
**6**	87	25	18
**7**	82	30	18
**8**	60	47	23
**9**	55	55	20
**10**	40	70	20
**11**	32	75	23
**12**	22	88	20

Number of subjects (n),

All infants were healthy at birth and none were on medications when sampled during the first month of life (month zero). During the first year of life, 19 of the infants had at some point used topical medication containing cortisone (for obstructive bronchitis, allergy or eczema); on average, three infants were treated each month. Other medications used by the infants included antibiotics, acetaminophen (paracetamol), non-steroidal anti-inflammatory drugs and nose drops for runny nose, as well as medication for bowel symptoms such as colic and constipation. A total of 45 infants were treated at some point with one of the non-cortisone containing medications described above; on average, eight infants each month (Table B in S2 File, Cortisol concentrations in infants with cortisone treatment and reference intervals for salivary cortisol concentrations for all infants with and without cortisone treatment.)

No infant or mother had any serious disease requiring hospitalization or intravenous drugs. Among the mothers, 15 were treated with cortisone-containing medication (14 topical, one systemic) at some time during the first year of the infant’s life; on average, five mothers each month. The mothers used 39 additional medications at some point during the first year of the infant’s life; on average, 15 mothers each month. The medications included antibiotics, acetaminophen (paracetamol), non-steroidal anti-inflammatory drugs and nose drops, as well as medication for bowel symptoms such as gastritis and constipation. Nine mothers used oral contraceptives and two took anti-depressant medication. Each of four mothers took one of the following medications: warfarin for thrombosis, immune globulin for IgA-deficiency, atenolol for hypertension and ferrous glycine sulfate for anemia.

The entire data set is provided as S1 File, Microsoft Excel file containing all original data on which the study is based.

### Ethical approval

All babies were recruited after delivery in the maternity ward. The local ethics committee at Linköping University approved the study (D# M196-06), and written informed consent was obtained from all parents.

### Saliva samples

To determine when full-term infants establish a CCR and to obtain reference material for salivary cortisol levels in infants, 8800 saliva samples were collected and analyzed to determine cortisol levels. The parents took the first samples in the home environment during the infant’s first or second week of life (mean age 10±4 days). Infants were required to be older than 2 days to avoid interference with post-delivery cortisol levels [16]. A nurse specially trained for the purpose instructed the parents on how to take all samples at home monthly during the first year (a total of thirteen sampling occasions). Earlier studies all confirm that the greatest differences in cortisol levels are early in the morning and late in the evening [3]; therefore, sampling took place at 07:30 in the morning and 19:30 in the evening. To create reference material, additional sampling was carried out between 10:00 and noon (sample times 07:30–09:30, 10:00–12:00 and 19:30–21:30 were accepted; samples collected outside these time limits were excluded). Samples were obtained on two subsequent days on the same day each month that the infant was born.

The parents were instructed to take the saliva samples at least one hour after the infant was fed solid food, 30 minutes after liquid food, one hour after they slept or cried, and one hour after riding in the car. Saliva samples were stored in the refrigerator until the parents mailed them (within one week) to the University Hospital in Linköping (salivary cortisol levels in samples have proven to be stable at room-temperature for at least two weeks prior to analysis [17]). Once the samples arrived at the laboratory they were centrifuged and stored at -70°C. A radioimmunoassay was used to analyze cortisol levels [13]. Samples were run in duplicate. Because of the numerous samples, all samples from each individual could not be analyzed in the same assay. Inter-assay coefficients of variation were 12% at 2.0 nmol/L and 6% at 10.0 nmol/L.

### Questionnaires

The Baby Behavior Questionnaire (BBQ), validated in a Swedish sample by Hagekull et al. in 1985, measures six dimensions—Intensity/Activity, Regularity, Approach/Withdrawal, Sensory Sensitivity, Attentiveness, and Manageability—based on a total of 31 items [18]. The six dimensions are usually presented separately [18]. In the current study, Regularity was measured using six items on a scale of increasing regularity from 1 to 5. The six items were expressed as: “Going to sleep at the same time,” “Waking up at the same time,” “Hungry at the same time,” “Eating the same amount of food every day,” “Taking a nap at the same time every day,” and “Having a regular bowel movement schedule.”

The Life Incidence of Traumatic Events checklist (LITE) is a validated questionnaire designed to detect trauma [19]. Parents were asked to fill out a Swedish translation of LITE (designed for infants) at birth (month zero), and at months one, six and twelve. The Swedish version has been used in earlier studies [20]. The LITE checklist consists of 15 items with fixed answers about lifetime occurrence of traumatic life events, such as “a family member was hospitalized (parent, sibling, grandparent, cousin, aunt or uncle),” “parents separated,” and “infant hurt or threatened.”

### Statistics

The software IBM SPSS Statistics, version 22 was used for statistical analysis.

CCR was defined as follows: the infants, on a group level in a specific month, had developed a cortisol circadian rhythm where the median cortisol morning level was significantly higher than the median cortisol evening level. Samples were obtained on two consecutive days each month, and prior to analysis, averages were calculated from these two morning and evening levels (for each infant and month), respectively. To avoid attributing higher weight to certain individuals in the analysis due to large inter-individual differences in absolute cortisol levels, an evening/morning cortisol index was created for each individual. The evening cortisol value was divided by the same day’s morning cortisol value for each individual. Morning and evening levels were subsequently compared using the Wilcoxon rank-sum test for each of the 13 months. By creating an evening/morning cortisol index for each individual, and using nonparametric statistics, potential outliers did not have to be excluded.

The Spearman rank correlation coefficient was used as measure of how well the relation between two variables can be described by a monotonic function. In this case—increase with age of the saliva concentrations of cortisol in the morning and decrease with age in the saliva concentrations in the evening.

In the material relating to individual CCR development during the infants’ first year of life, a cut-off of a 20%-difference was adopted. This cut-off limit was based on the accuracy of the method and is in line with cut-off limits used in other studies [8, 10, 21].

The hypothesis that there would be a correlation between the BBQ Regularity item and the development of CCR was tested using Spearman’s correlation analysis to compare the BBQ Regularity item with the morning-evening salivary cortisol index. The possible effect of trauma (as measured by LITE) on the development of CCR was assessed using the same principles.

The impact of samples with a macroscopically abnormal appearance, such as different color than the typical saliva sample, was tested by comparing all samples with an abnormal appearance with all samples with normal appearance using the Wilcoxon rank-sum test. Results from this group of samples with abnormal appearance showed no statistically significant difference from the results of the entire group and therefore were not excluded from the final statistical analyses. The same principles were adopted for the following potential confounders: a) infants with topical cortisone medication, b) infants with non-cortisone containing medication, c) infants of mothers using cortisone medication (topical or systemic), d) infants of mothers using non-cortisone containing medication. Results from these four groups of infants did not show any statistically significant difference from the results of the entire group and were therefore included in the final statistical analyses.

Salivary cortisol concentrations are presented both as monthly median (quartile 1–quartile 3) and mean (standard deviation).

### Protocol violations—missing data

In the current study, cortisol values were lost to analysis due to either of the following two reasons: 1) the samples were not sent to the hospital by the parents or 2) the samples contained too little saliva for satisfactory analysis. If an infant lacked one of the two samples for a specific month and time-point, the other was used for calculating the morning/evening ratio. If an infant lacked both samples for a specific month and time-point, no evening/morning ratio could be calculated. Missing cortisol data is presented in Table C in S2 File, in absolute numbers and as a percentage of the planned 260 samples for each month and time of day (130 infants taking samples from two consecutive days = 260 samples). In total, 1348 (13.3%) of 10140 samples were lost (Table A in S2 File, Protocol violations—missing cortisol data).

## Results

### Development of CCR

**Fig 1:**
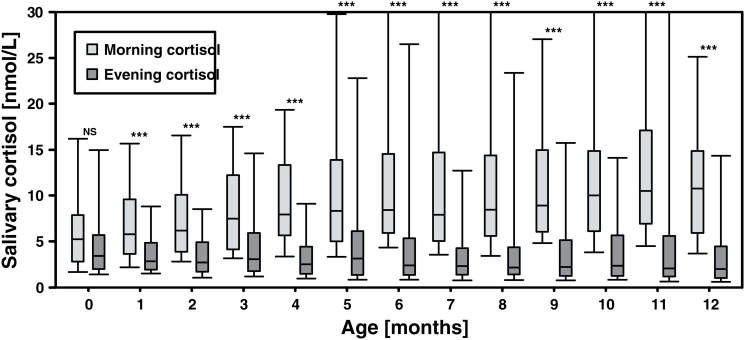
Light grey box plots: monthly morning median cortisol value, inter quartile range one and three. Dark grey box plots: monthly evening cortisol median value, inter quartile range one and three. *** = P<0.000 significant difference between evening and morning cortisol, Wilcoxon’s rank-sum test, month one to twelve.

**Fig 2:**
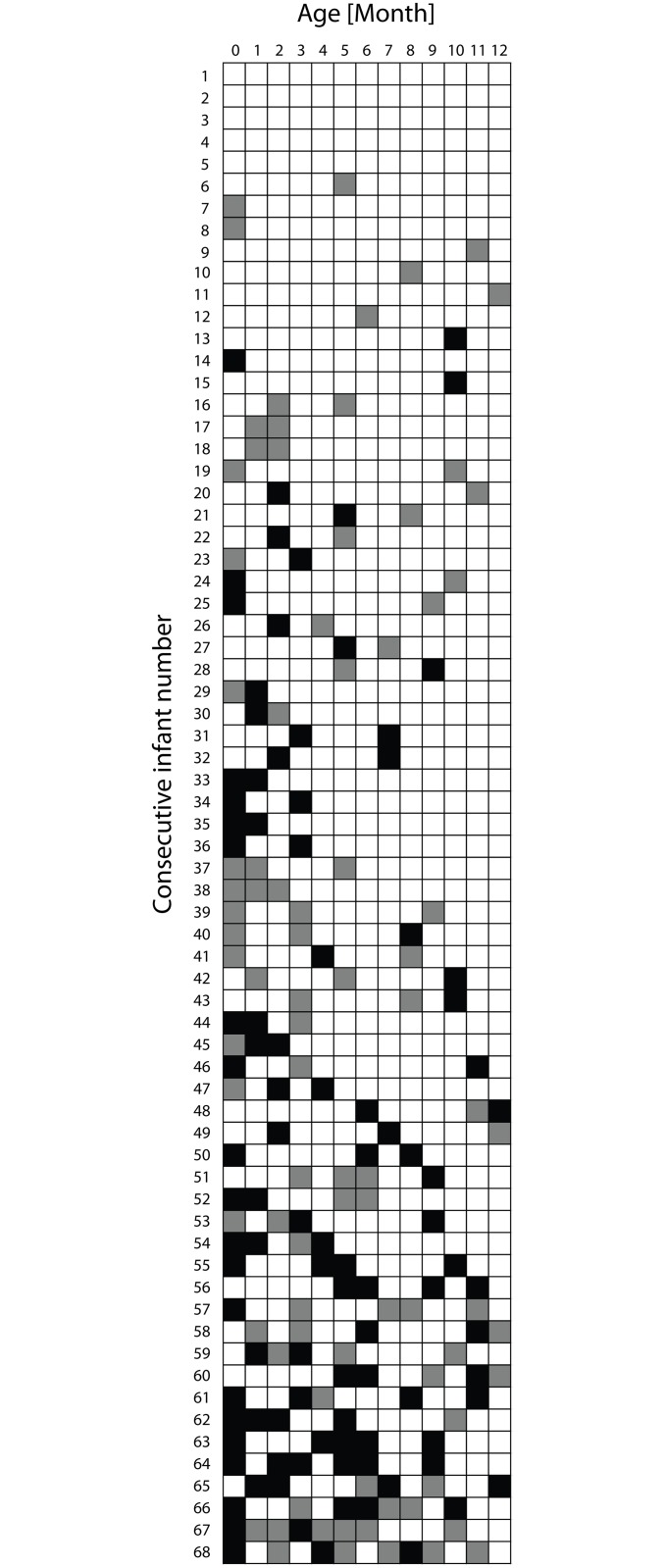
Individual CCR development, arbitrarily defined based on accuracy of the method, in the 68 infants with analyzed morning and evening cortisol concentrations for all 13 months. White squares: "CCR positive" (Ratio: evening/morning cortisol <0.80). Black squares: "CCR negative" (Ratio: evening/morning cortisol >1.20). Gray squares: neither "CCR positive" nor "CCR negative" (Ratio: evening/morning cortisol 0.80–1.20). One column represent each month. Each row represents one infant (68 infants).

On a group level, from one month on infants developed a CCR with a significant difference between morning and evening cortisol levels, statistically persistent throughout the first year of life (Fig 1; Table 3). Fig 2 presents individual patterns of CCR development, according to an arbitrary cut-off of a 20%-difference, in the 68 infants with analyzed morning and evening cortisol concentrations for all 13 months. Even though Fig 2 displays a general pattern of successively increasing CCR stability, coherent with the analyses presented in Fig 1, it also visualize a considerable individual variability.

**Table 3:** Monthly difference between evening and morning salivary cortisol levels.

Age at sampling, (Month)	Age at sampling, days Mean (SD)	Subjects with analyz-able samples (n)	Difference evening/morning cortisol value P-value
E	10(4)	95	0.226
1	35(4)	120	0.000
2	64(5)	121	0.000
3	94(5)	114	0.000
4	123(5)	118	0.000
5	155(5)	111	0.000
6	185(5)	112	0.000
7	215(5)	112	0.000
8	246(5)	107	0.000
9	275(4)	105	0.000
10	306(5)	107	0.000
11	338(6)	106	0.000
**12**	**367(5)**	**107**	**0.000**

Monthly p-values for difference between evening and morning cortisol on a group level using Wilcoxon’s rank-sum test. Age for sampling measured in: Month and Mean Day for sampling during full-term infants’ first year of life. Number of infants that submitted analyzable samples for evening/morning range in each month.

Excluding samples from infants on cortisone medication did not alter the results, as presented in (Table B in S2 File).

### Questionnaires

The BBQ Regularity item did not increase over the months covered by the study (Table 4).

**Table 4:** “Regularity” measured with The Baby and Behavior Questionnaire.

Regularity			
**Month**	1	6	12
**Mean**	3.51	2.52	2.14
**SD**	0.84	0.73	0.59

Scale 1–5 where increased numbers represent increased regularity. Standard deviation (SD), Baby and Behavior Questionnaire (BBQ).

No significant correlation was observed between the BBQ Regularity item score and the development of CCR (Table 5).

**Table 5:** Correlation between salivary cortisol evening/morning quotients and Regularity and Trauma, respectively.

BBQ			LITE		
Month	n =	Correlation coefficient	Month	n =	Correlation coefficient
			0	94	0.003
1	87	0.02	1	119	0.181
6	91	0.054	6	109	0.082
12	99	0.166	12	107	0.008

Spearman’s analysis for correlation between the Baby Behavior Questionnaire (BBQ) Regularity item and the salivary cortisol evening/morning quotient and Spearman’s analysis for correlation between traumas detected by the Life Incidence of Traumatic Events checklist (LITE) and the salivary cortisol evening/morning range is presented in column three.

### Baby and Behavior Questionnaire (BBQ). Life Incidence of Traumatic Events checklist (LITE)

Similarly, no significant correlation was found between trauma, as tested by LITE, and cortisol levels (Table 5). Too few traumas occurred to allow checking for possible multiple trauma influences (Table 6).

**Table 6:** Number of reported traumas at four-month periods during infants’ first year of life.

Number of Traumas	Month 0	Month 1	Month 6	Month 12
**1**	36	15	19	19
**2**	7	1	2	3
**3**	0	0	0	0
**4**	1	0	0	0

During month zero the mothers answered questions about the number of traumas during their entire pregnancy, which resulted in a larger number of traumas because of the longer time period. The traumas were otherwise very few in the group and often milder in nature. The most common trauma involved hospitalization of a family member (including grandfather, cousin or sister).

### Reference intervals

Table 7 presents median and mean morning, noon and evening cortisol concentrations.

**Table 7:** Reference intervals for salivary cortisol [nmol/L].

Age	Morning values: 07:30–09:30				Noon values: 10:00–12:00				Evening values: 19:30–21:30			
ee	Median	Q1-Q3	Mean	SD	Median	Q1-Q3	Mean	SD	Median	Q1-Q3	Mean	SD
**0**	5.1	2.8–8.2	10.2	21.0	5.0	3.7–8.6	8.5	11.8	3.4	2.1–5.7	7.5	15.3
**1**	5.8	3.7–9.8	8.3	8.3	4.8	2.8–6.8	6.6	7.5	2.8	1.9–4.8	5.1	6.9
**2**	6.1	3.9–9.8	8.0	7.4	5.2	3.4–7.3	6.3	5.8	2.7	1.7–4.9	4.4	5.9
**3**	7.5	4.2–11.9	13.8	38.0	5.9	4.1–8.5	9.1	17.1	3.3	1.9–5.9	9.2	22.5
**4**	8.0	5.7–13.5	12.6	18.2	6.2	3.7–9.0	8.5	15.0	2.5	1.5–4.4	6.8	17.9
**5**	8.3	5.0–13.8	16.9	35.5	5.9	3.5–8.6	16.6	43.3	3.1	1.3–6.2	12.9	32.0
**6**	8.9	6.2–14.9	24.6	63.8	5.4	3.9–8.8	20.3	77.1	2.3	1.3–5.0	19.4	81.4
**7**	7.7	5.0–14.6	26.5	78.9	5.1	3.6–8.1	21.5	71.0	2.3	1.4–4.2	10.9	35.1
**8**	8.4	5.6–14.2	33.0	95.4	5.9	3.5–10.6	22.9	77.9	2.2	1.4–4.3	20.3	80.6
**9**	8.9	6.0–14.8	21.9	49.4	5.6	3.6–9.9	19.4	77.6	2.3	1.2–5.2	9.8	28.5
**10**	10.0	6.1–14.2	21.3	43.6	5.4	3.7–8.9	14.4	34.3	2.4	1.3–5.6	10.7	28.7
**11**	10.5	6.9–17.1	23.9	60.7	5.3	3.4–9.7	11.9	22.1	2.1	1.2–5.6	13.4	40.6
**12**	10.9	5.9–14.4	22.9	54.4	5.2	3.1–8.6	14.4	35.4	2.0	1.1–4.2	11.6	33.9

Monthly median (quartile 1–quartile 3) and mean (standard deviation) salivary cortisol levels [nmol/L] at three different sampling times: morning (07:30–09:30), noon (10:00–12:00) and evening (19:30–21:30).

Morning median levels steadily increased throughout the first year of life (Spearman 0.248, p<0.01) with a parallel decrease in evening levels (Spearman -0.128, p<0.01), illustrating a continuously more pronounced CCR for each month during the first year of life. Noon median and mean levels remained fairly consistently between morning and evening levels.

## Discussion

The current results, based on 8800 samples from 130 healthy full-term infants, show that CCR is established at one month of age. We also present individual patterns of CCR development throughout the entire first year for 68 infants, which is also unparalleled in the literature. The monthly salivary cortisol levels that we presented may be used as reference intervals for salivary cortisol in full-term infants up to the age of one year.

Seven earlier studies investigating the development of CCR in full-term infants have been published to date. Results pertaining to the development of CCR have been highly divergent, ranging from 7.4 weeks up to more than 9 months for the appearance of CCR [4–10]. One possible explanation for such discrepant results is that saliva samples were collected at different ages. Our sampling times and CCR results were similar to the studies presented by Price (1983), Santiago (1996) and Custodio (2007), while other studies did not include sampling during the first month of life [7, 9]. Two of the previous studies only included single observations, risking unreliable conclusions [5, 6]. The primary aim of another study was to investigate cortisol response to vaccination; i.e., it was not designed to explore CCR development [7]. Another frequently-cited study in the field presented salivary cortisol levels of preterm infants; since our results are based on full-term infants, comparisons are unwarranted [21]. Our study reaches a different conclusion than the studies by Santiago et al. and Custodio et al. [8, 10]. These studies, which were performed according to protocols comparable to that of the current study, reported that CCR developed at 8 weeks of age and 7.4–7.8 weeks of age, respectively. These differences may be due to the number of subjects used in the studies. Our study included 130 infants throughout an entire year, while the largest earlier study included at most 34 infants with sampling until six months of age [10]. Clearly, the larger the number of included individuals, the greater the statistical power to detect signs of CCR development. Notably, the earlier study with the highest number of individuals—34 full-term twin infants—presented the results that are most similar to our study [10].

Due to large inter-individual variation in absolute morning-evening cortisol differences, the Wilcoxon rank-sum test was selected for the main tests in the current study, as with earlier studies in the field [8, 10, 21]. Also, several previous studies have corroborated that absolute basal cortisol levels vary substantially both intra-individually and inter-individually [22], which was why an evening/morning cortisol index for each individual was considered more appropriate when analyzing data from multiple individuals in the current study. Price and coworkers analyzed their data with parametric tests and defined CCR as occurring when the morning cortisol level was 2 nmol/L higher than evening cortisol level [4, 9]. This approach seems suboptimal due to large inter-individual differences in absolute cortisol levels, making a 2 nmol/L difference proportionally extremely large for some individuals, while small for others.

The sample of children is not a random sample from the population but rather a “convenience sample” as consecutive parents were asked to participate and the newborns of those willing to take on this considerable task were included. The sample included a higher portion of parents with university degrees than the general population.

Even though a steady CCR was established during the first year of life, no regularity (as described in the BBQ Regularity item) developed. The observed lack of correlation between hormonal rhythm and behavioral regularity may be due to insufficient sensitivity in the BBQ questionnaire. However, since the questionnaire was designed and validated for regularity purposes, a more plausible interpretation may simply be that there is no correlation between early development of CCR and behavioral regularity. An earlier study [4] found no statistically significant association between sleeping pattern regularity and development of CCR, though six out of eight infants developed CCR *after* they started sleeping through the night.

Even though the median only moderately increased throughout the year, the 90th percentile increased more steeply, especially from month 5, as seen in Fig 1. Regarding the reason for this increased dynamic, one can only speculate. Possibly, the maturing HPA-axis becomes increasingly responsive to stressors, thereby causing higher peak values in comparison to the median.

No significant correlation was observed between the possible confounding factor trauma and the development of CCR. Previous studies have shown that traumas, especially multiple traumas in abused adolescents, influence salivary cortisol levels [11]. A plausible explanation for the lack of significant association between CCR and trauma in the current study may be that very few traumatic life events have occurred in this healthy full-term infant population.

### Limitations

One limitation of the current study which it shares with practically all similar studies is that it included only healthy full-term infants. It would be of substantial importance to study the development of CCR e.g. in preterm infants, infants with illnesses or diseases requiring hospitalization. The sampling frequency is also a limitation. Cortisol concentrations are highest in the morning and reach their nadir in the evening. Our sampling times were morning, noon and evening, but the cortisol pattern over an entire 24-hour period may yield even more information about cortisol circadian rhythm. Further, the date of birth could possibly influence the development of CCR. In the current study, 44 of the infants were born in the three darkest months (November to January), 11 in the three brightest months (May to July) and 74 in the remaining six spring and fall months. This slight bias toward births in the darkest month was totally accidental, since infants were recruited all year round for almost four years, but is a possible source of error to be kept in mind when assessing the results. There is a relation between regularity and patterns of sleep and cortisol concentrations including the cortisol awakening response. It is a limitation of the present study that studying cortisol concentration regularly throughout the entire first year of life was given precedence over the study of the relation between cortisol concentrations and sleep.

### Conclusions

Cortisol circadian rhythm in infants can already be detected—on a group level—at one month of age, which is earlier than previous studies have shown. Since the main focus of the current study was to detect circadian rhythm on a group level, an interesting avenue for future research would be to explore a more detailed analysis on the individual level, which could be accomplished by obtaining daily salivary cortisol measurements throughout the first two months of life, in order to more accurately pinpoint individual differences in cortisol rhythm development. The current study also provides salivary cortisol reference intervals for healthy, full-term infants during the first year of life.

## Supporting Information

S1 File A Microsoft Excel—file containing all original data on which the study is based. (XLSX)

S2 File A Microsoft Word—file containing three tables: Reference intervals for salivary cortisol concentrations [nmol/L] for all infants with and without cortisone treatment (Table A), Cortisol concentrations [nmol/L] in infants with cortisone treatment (Table B), Protocol violations—missing cortisol data (Table C). (DOCX)
