# Correction: Development of Salivary Cortisol Circadian Rhythm and Reference Intervals in Full-Term Infants

**DOI:** 10.1371/journal.pone.0151888

**Published:** 2016-03-14

**Authors:** Katrin Ivars, Nina Nelson, Annette Theodorsson, Elvar Theodorsson, Jakob O. Ström, Evalotte Mörelius

In Table 4, the values for mean and standard deviation are incorrect. Please see the corrected Table 4 here.

**Table 4:** “Regularity” measured with The Baby and Behavior Questionnaire.

Regularity			
**Month**	1	6	12
**Mean**	2.5	3.5	3.9
**SD**	0.9	0.7	0.6
**p-value for possible differences between months**		0.000*	0.000**

Scale 1–5 where increased numbers represent increased regularity. Standard deviation (SD), Baby and Behavior Questionnaire (BBQ).

*Between month one and six

** Between month six and twelve

In Table 5, the values for Correlation coefficient and n for BBQ are incorrect. Please see the corrected Table 5 here.

**Table 5:** Correlation between salivary cortisol evening/morning quotients and Regularity and Trauma, respectively.

BBQ			LITE		
Month	n =	Correlation coefficient	Month	n =	Correlation coefficient
			0	94	0.003
1	84	0.048	1	119	0.181
6	88	-0.047	6	109	0.082
12	96	-0.122	12	107	0.008

Spearman’s analysis for correlation between the Baby Behavior Questionnaire (BBQ) Regularity item and the salivary cortisol evening/morning quotient and Spearman’s analysis for correlation between traumas detected by the Life Incidence of Traumatic Events checklist (LITE) and salivary cortisol evening/morning quotient is presented in column three.

There is an error in the first sentence of the “Questionnaires” subsection of the Results. The correct sentence is: The BBQ Regularity item increased significantly over the months covered by the study (Table 4).

There is an error in the first sentence of the fifth paragraph of the Discussion. The correct sentence is: A steady CCR was established during the first year of life as well as a significant increase in regularity (as described in the BBQ Regularity item) developed (Table 4).
