# Supplementary material for: Development of Salivary Cortisol Circadian Rhythm and Reference Intervals in Full-Term Infants
Source: PLoS One. 2015 Jun 18;10(6):e0129502. doi: 10.1371/journal.pone.0129502 (PMC4472813; doi:10.1371/journal.pone.0129502)
Supplement: S2 File — (DOCX) [file pone.0129502.s002.docx]

# Supporting information tables

**Supplement Table A.** Reference intervals for salivary cortisol concentrations [nmol/L] for all infants with and without cortisone treatment

|  | **All infants** | | | | | | **Cortison treated infants excluded** | | | | | |
| --- | --- | --- | --- | --- | --- | --- | --- | --- | --- | --- | --- | --- |
| **Month** | **Number** | **Morning** | | **Evening** | | **P-value** | **Number** | **Morning** | | **Evening** | | **P-value** |
|  |  | Median | Q1 - Q3 | Median | Q1 - Q3 |  |  | Median | Q1 - Q3 | Median | Q1 - Q3 |  |
| **3** | 114 | 7.5 | 4.2 - 11.9 | 3.3 | 1.9 - 5.9 | 0.000 | 111 | 7.5 | 4.2 - 11.9 | 3.1 | 1.8 - 5.8 | 0.000 |
| **4** | 118 | 8.0 | 5.7 - 13.5 | 2.5 | 1.5 - 4.4 | 0.000 | 117 | 7.9 | 5.7 - 13.2 | 2.5 | 1.5 - 4.4 | 0.000 |
| **5** | 111 | 8.3 | 5.0 - 13.8 | 3.1 | 1.3 - 6.2 | 0.000 | 109 | 8.0 | 5.0 - 12.7 | 3.1 | 1.3 - 5.5 | 0.000 |
| **6** | 112 | 8.9 | 6.2 - 14.9 | 2.3 | 1.3 - 5.0 | 0.000 | 109 | 8.8 | 6.0 - 14.6 | 2.2 | 1.3 - 4.6 | 0.000 |
| **7** | 112 | 7.7 | 5.0 - 14.6 | 2.3 | 1.4 - 4.2 | 0.000 | 109 | 7.6 | 5.0 - 13.8 | 2.3 | 1.4 - 4.0 | 0.000 |
| **8** | 107 | 8.4 | 5.6 - 14.2 | 2.2 | 1.4 - 4.3 | 0.000 | 104 | 8.2 | 5.6 - 13.8 | 2.1 | 1.4 - 4.1 | 0.000 |
| **9** | 105 | 8.9 | 6.0 - 14.8 | 2.3 | 1.2 - 5.2 | 0.000 | 102 | 8.7 | 6.0 - 14.4 | 2.2 | 1.2 - 4.9 | 0.000 |
| **10** | 107 | 10.0 | 6.1 - 14.2 | 2.4 | 1.3 - 5.6 | 0.000 | 103 | 9.9 | 6.1 - 13.9 | 2.2 | 1.3 - 5.0 | 0.000 |
| **11** | 106 | 10.5 | 6.9 - 17.1 | 2.1 | 1.2 - 5.6 | 0.000 | 104 | 10.4 | 6.8 - 16.5 | 2.1 | 1.2 - 5.3 | 0.000 |
| **12** | 107 | 10.9 | 5.9 - 14.4 | 2.0 | 1.1 - 4.2 | 0.000 | 103 | 10.6 | 5.9 - 14.0 | 1.9 | 1.0 - 4.0 | 0.000 |

The All infants columns include a total of 19 cortisone treated infants contributing with a total of 28 measurement in months 3-12. No infants were subjected to cortisone treatments months 0-2, and these months are therefore not presented in the table. P-values are for differences between morning and evening values.

**Supplement Table B.** Cortisol concentrations [nmol/L] in infants with cortisone treatment

| **Month** | **Infant code** | **Morning cortisol concentration** | **Evening cortisol concentration** | **Evening/Morning-Ratio** |
| --- | --- | --- | --- | --- |
| **3** | 33 | 385.8 | 184.8 | 0.5 |
| **3** | 116 | 4.1 | 2.8 | 0.7 |
| **3** | 153 | 5.2 | 9.6 | 1.8 |
| **4** | 156 | 10.6 | 4.4 | 0.3 |
| **5** | 126 | 29.8 | 127.9 | 4.3 |
| **5** | 143 | 43.4 | 55.5 | 1.3 |
| **6** | 32 | 141.2 | 28.7 | 0.2 |
| **6** | 62 | 7.4 | 34.5 | 4.7 |
| **6** | 126 | 559.0 | 750.0 | 1.3 |
| **7** | 36 | 150.0 | 150.0 | 1.0 |
| **7** | 126 | 750.0 | 150.0 | 0.2 |
| **7** | 156 | 22.7 | 7.1 | 0.3 |
| **8** | 17 | 405.6 | 104.3 | 0.3 |
| **8** | 117 | 466.6 | 145.5 | 0.3 |
| **8** | 126 | 750.0 | 750.0 | 1.0 |
| **9** | 28 | 249.6 | 111.9 | 0.4 |
| **9** | 32 | 24.1 | 3.2 | 0.1 |
| **9** | 113 | 36.6 | 5.4 | 0.1 |
| **10** | 36 | 288.3 | 150.0 | 0.5 |
| **10** | 64 | 251.1 | 14.0 | 0.1 |
| **10** | 113 | 84.8 | 26.0 | 0.3 |
| **10** | 126 | 150.0 | 102.7 | 0.7 |
| **11** | 14 | 24.0 | 336.3 | 14.0 |
| **11** | 126 | 150.0 | 150.0 | 1.0 |
| **12** | 8 | 91.9 | 236.4 | 2.6 |
| **12** | 36 | 142.1 | 86.9 | 0.6 |
| **12** | 141 | 11.2 | 28.1 | 2.5 |
| **12** | 151 | 3.7 | 2.6 | 0.7 |

**Supplement Table C. Protocol violations – missing cortisol data.**

| Month | Number of missing cortisol samples (percent of planned 260 samples) | | |
| --- | --- | --- | --- |
|  | Morning | Noon | Evening |
| 0 | 58 (22%) | 48 (18%) | 50 (19%) |
| 1 | 12 (5%) | 18 (7%) | 14 (5%) |
| 2 | 14 (5%) | 26 (10%) | 14 (5%) |
| 3 | 26 (10%) | 24 (9%) | 26 (10%) |
| 4 | 22 (8%) | 24 (9%) | 24 (9%) |
| 5 | 34 (13%) | 40 (15%) | 36 (14%) |
| 6 | 28 (11%) | 28 (11%) | 36 (14%) |
| 7 | 30 (12%) | 34 (13%) | 36 (14%) |
| 8 | 40 (15%) | 42 (16%) | 44 (17%) |
| 9 | 44 (17%) | 42 (16%) | 46 (18%) |
| 10 | 42 (16%) | 42 (16%) | 46 (18%) |
| 11 | 44 (17%) | 46 (18%) | 46 (18%) |
| 12 | 40 (15%) | 42 (16%) | 42 (16%) |
